# Bilateral femoral neck fractures due to transient osteoporosis of pregnancy: a case report

**DOI:** 10.1186/1757-1626-1-120

**Published:** 2008-08-21

**Authors:** Charles A Willis-Owen, Jas S Daurka, Alvin Chen, Angus Lewis

**Affiliations:** 1 Dept Orthopaedics, Charing Cross Hospital, Fulham Palace Road, London, UK

## Abstract

We describe a case of bilateral femoral neck fractures secondary to transient osteoporosis of pregnancy, which were diagnosed after delivery due to the desire to avoid ionising radiation. These fractures were presumed to be secondary to transient osteoporosis of pregnancy and were treated successfully with internal fixation despite delayed presentation. We discuss the role of MRI in the evaluation of hip pain in pregnancy.

## Introduction

Transient osteoporosis of pregnancy (TOP) is a rare, idiopathic self-limiting condition typically associated with the third trimester of pregnancy. It almost always affects a single hip although bilateral presentation and involvement of the knee have been reported [1-3].

TOP usually presents with a sudden, quite severe onset of unilateral groin pain with no history of trauma. The patient may be unable to walk, or may have an antalgic gait. Pain is elicited by hip rotation, although a full range of motion is common. Radiographs are avoided in pregnancy where possible, and are a poor investigation for demonstrating early osteopaenia. Magnetic Resonance Imaging (MRI) reveals low signal intensity of bone marrow on T1 weighted images, and high signal on T2 weighted images suggestive of bone marrow oedema[4]. The natural history is of resolution of symptoms over the course of 3 to 6 months

Hip fracture secondary to TOP is very rare with only 12 reported patients in the literature to date; in two cases the hip fractures were bilateral[2,3,5-8]. The majority of these fractures were caused by a traumatic event. Atraumatic hip fractures secondary to TOP are even more unusual and are easily overlooked and hence may present to the orthopaedic surgeon at a late stage, making management more challenging.

We report of a case of bilateral TOP leading bilateral atraumatic femoral neck fractures that were diagnosed post partum. Despite the delay in presentation internal fixation was successfully carried out. We highlight the importance of adequate investigation of hip pain during pregnancy and discuss the role of MRI.

## Case presentation

A 34 year old Persian woman, gravida 1, para 0, presented at 22 weeks of pregnancy with a two week history of left hip pain with no apparent precipitating event. Her past medical history included mild Multiple Sclerosis from which she was asymptomatic. She did not smoke or drink alcohol, had no history of corticosteroid, anticonvulsant or anticoagulant use and was not on any other medication. Clinical examination was unremarkable and no investigations were deemed appropriate. The working diagnosis at this stage was non-specific hip pain related to pregnancy and supportive measures were instituted.

Over the following 12 weeks her hip pain worsened, and she started to experience pain in the contra lateral hip. Again there was no history of a traumatic event. Because of her pregnancy imaging of her hips was avoided. By 36 weeks of pregnancy she was unable to weight bear and became wheelchair bound. Pain in her hips and limitation of motion meant that a normal vaginal delivery was impossible; hence she underwent a caesarean delivery of a healthy baby at full term.

**Figure 1:**
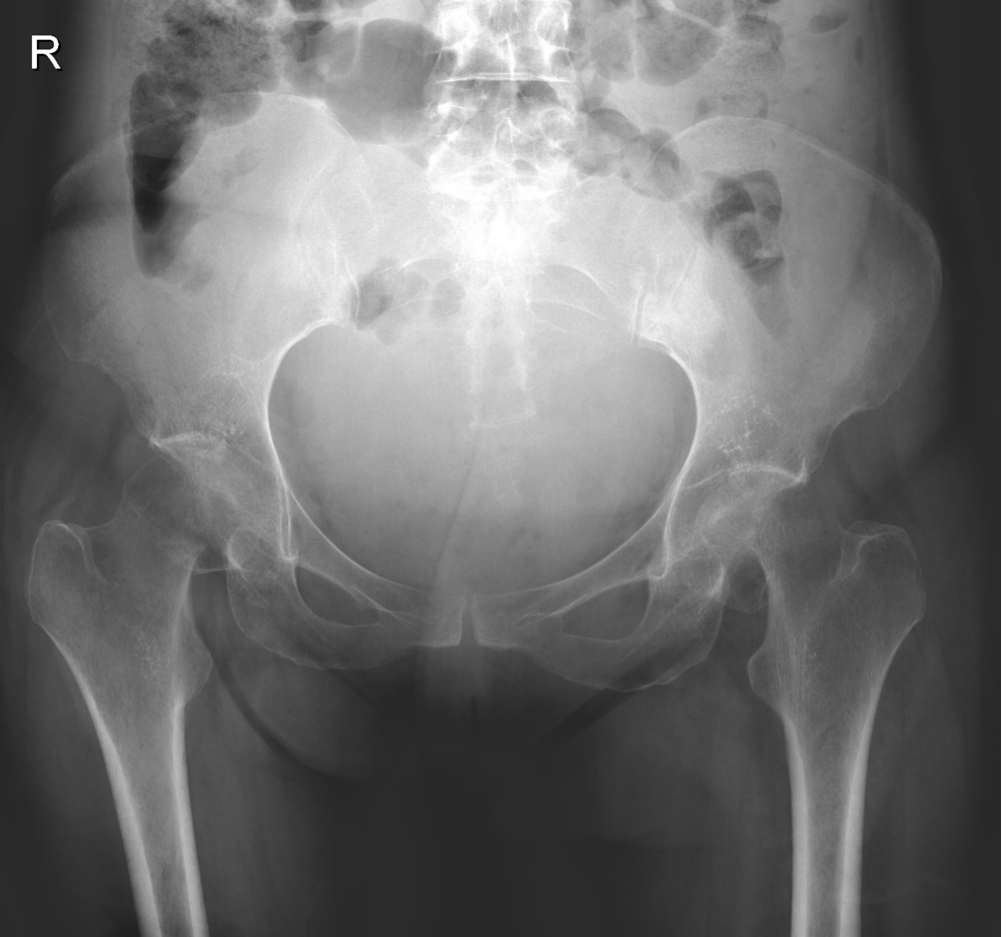
Antero-posterior radiograph of the pelvis post partum.

**Figure 2:**
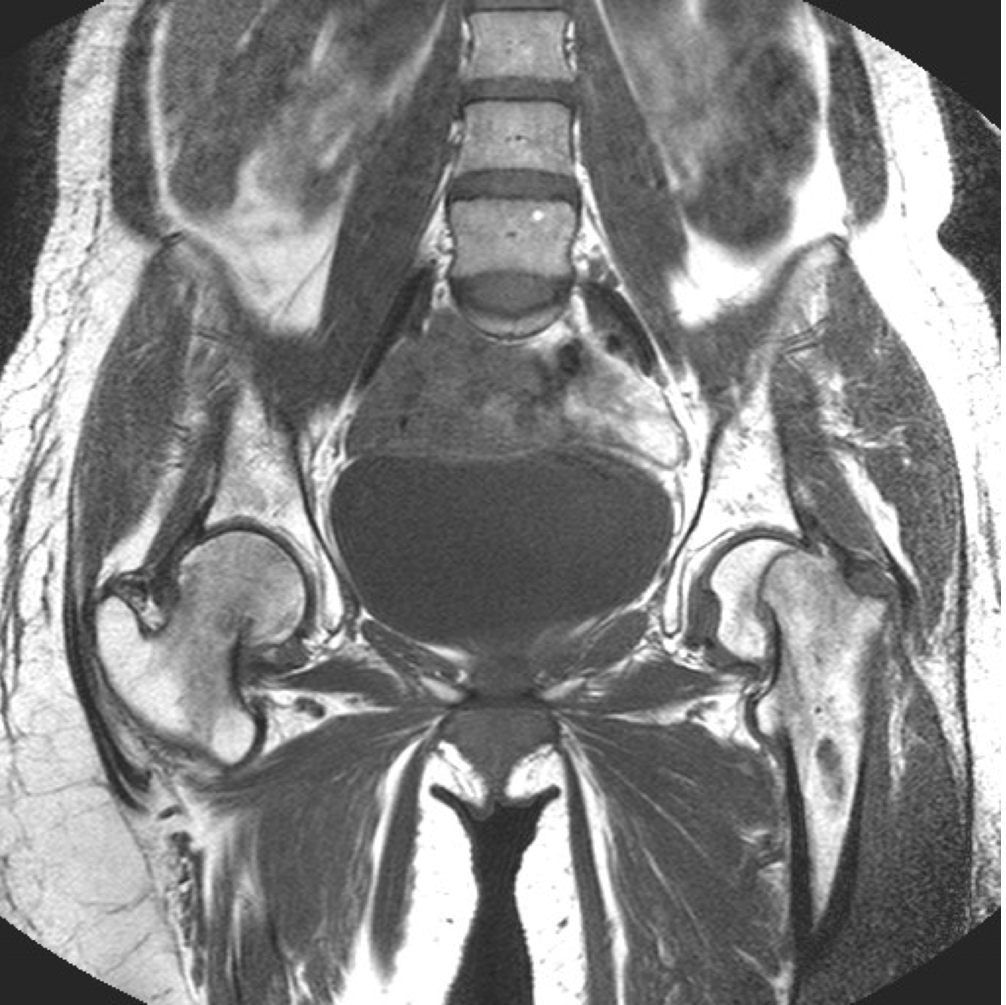
T1 weighted coronal MRI scan of the pelvis post partum.

She was brought to the attention of the orthopaedic team when plain radiographs (see figure 1) following delivery revealed a displaced intracapsular femoral neck fracture on the left and a valgus impacted right intracapsular femoral neck fracture on the right. The radiographs also revealed considerable osteopaenia. MRI (see figure 2) revealed these fractures, with reduced signal on T1 and increased signal on T2 in the femoral necks in keeping with TOP.

**Figure 3:**
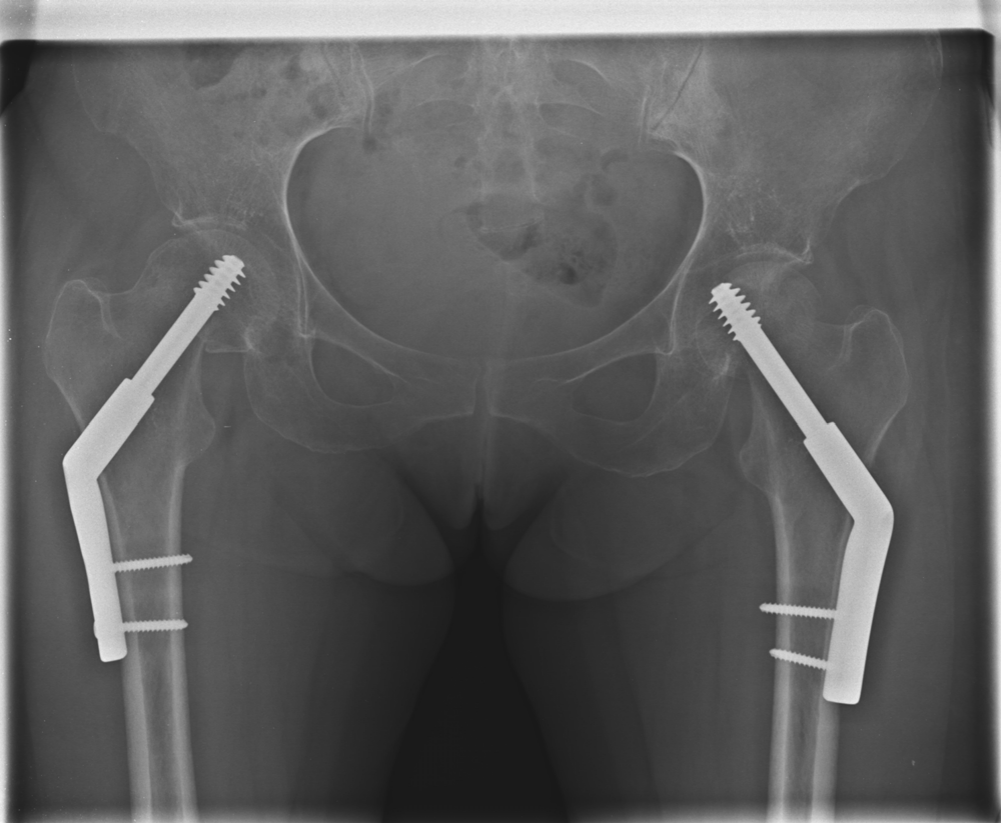
Antero-posterior radiograph of the pelvis post fixation with dynamic hip screws.

She underwent closed reduction and internal fixation of the left hip. The right hip was internally fixed in situ. Two hole 135 degree dynamic hip screws were used in order to provide sufficient stability to allow immediate mobilisation despite bilateral fractures. Difficulty was encountered in ensuring that the threads of the dynamic hip screw had crossed the fracture site in the left hip as the level of the fracture was high in the femoral neck, consequently the tip of the implant had to be implanted close to the subchondral plate (see figure 3).

The post-operative course was uncomplicated and the hip pain significantly improved immediately. Full weight bearing on the right, and partial weight bearing on the left was initiated on the first postoperative day, and maintained for the first 12 weeks. Check radiographs at 3 months showed no loss of fixation and the fractures appeared to be uniting in an adequate position. At six months she was pain free with no evidence of avascular necrosis or implant failure.

## Discussion

Musculoskeletal complaints are very common in pregnancy. The position and weight of the gravid uterus alters the centre of gravity and loading patterns of the axial and appendicular skeleton, whilst hormonal changes lead to joint laxity, and fluid retention may cause neural compression[9]. The majority of musculoskeletal complaints are not serious, and are managed conservatively without a specific diagnosis.

Pregnant women frequently complain of hip or pelvic pain. The differential diagnosis includes some serious problems that need to be excluded, namely transient osteoporosis, osteonecrosis and pubic symphysiolysis.

Conventionally ionising radiation is avoided during pregnancy although Brodell *et al. *suggested that in the third trimester of pregnancy the benefits of adequate investigation of hip pain may outweigh the minimal risks[5]. There is no conclusive evidence that MRI has deleterious effects, however the safety of MRI has yet to be definitively proven[10]. It is in common use in the third trimester of pregnancy where clinically indicated[11] and is generally considered to be safe[12]. MRI has a high sensitivity for diagnosis of occult hip fracture[13] and can reliably distinguish between osteonecrosis transient osteoporosis[4], making it the investigation of choice for hip pain in the third trimester of pregnancy.

Displaced intracapsular fractures have a high incidence of non-union and avascular necrosis[14]. It has however been shown that the risk of non-union is independent of bone quality[15] therefore in young patients with high value hips internal fixation should be the goal.

## Conclusion

This case report highlights the need for vigilance in the assessment of musculoskeletal complaints in pregnancy, and demonstrates that the more conservative approach of internal fixation is viable. We suggest MRI should be considered for women presenting with significant hip pain in the third trimester of pregnancy.

## Consent

Written informed consent was obtained from the patient for publication of this case report and accompanying images. A copy of the written consent is available for review by the Editor-in-Chief of this journal.

## Competing interests

The authors declare that they have no competing interests.

## Authors' contributions

AC assessed the patient at first presentation, JD and AL carried out the surgery, CWO was involved throughout admission and follow-up and was the major contributor in writing the manuscript. All authors contributed to, read and approved the final manuscript.
